# Clinical efficacy of Qi Di laxative decoction in the treatment of functional constipation: A protocol for systematic review and meta-analysis

**DOI:** 10.1097/MD.0000000000023806

**Published:** 2020-12-18

**Authors:** Jian-Fang Wang, Zhong-Mei Sun, Jun-Xiang Li

**Affiliations:** a Dongfang Hospital of Beijing University of Chinese Medicine; b Beijing University of Chinese Medicine, Beijing, China.

**Keywords:** functional constipation, protocol, Qi Di laxative decoction, traditional Chinese medicine

## Abstract

**Background:**

Functional constipation (FC) is a common gastrointestinal disorder characterized by slow bowel movement and defecation difficulties, significantly impacting patients’ quality of life and exerting heavy financial burden to whole society. However, more than 50% FC patients are not completely satisfied with current therapies and alternative therapies are urgently required. Increasing evidences have demonstrated that traditional Chinese medicine has a good therapeutic effect on FC, which is well known for its multitarget and multimode effects on diverse diseases as well as less side effects. Furthermore, studies proved that Qi Di Laxative Decoction was an effective treatment for FC. Its safety and effectiveness should be verified by further studies.

**Methods:**

We will search the following electronic databases for randomized controlled trials to evaluate the clinical efficacy of Qi Di Laxative Decoction in treating FC: Wanfang and Pubmed Database, China National Knowledge Infrastructure Database, Cochrane Central Register of Controlled Trials, Cumulative Index of Nursing and Allied Health Literature, and Excerpta Medica database. Each database will be searched from inception to November 2020. The entire process will include study selection, data extraction, risk of bias assessment, and meta-analyses.

**Results:**

This proposed study will evaluate the clinical efficacy of Qi Di Laxative Decoction for patients with FC. The outcomes will include changes in FC relief and adverse effect.

**Conclusion:**

This proposed systematic review will evaluate the existing evidence on the clinical efficacy of Qi Di Laxative Decoction in treating FC.

**Dissemination and ethics:**

The results of this review will be disseminated through peer-reviewed publication. Because all of the data used in this systematic review and meta-analysis has been published, this review does not require ethical approval. Furthermore, all data will be analyzed anonymously during the review process.

**OSF Registration Number:**

DOI 10.17605/OSF.IO/M2ESR

## 1 Introduction

Functional constipation (FC) is a common gastrointestinal disorder characterized by slow bowel movement and defecation difficulties, affecting over 14.0% adults worldwide.^[1,2]^ Female gender, old age, and low socioeconomic status are risk factors for people to have FC. FC significantly impacts patients’ quality of life and exerts heavy financial burden to whole society.^[3–5]^ People with mild or moderate FC can be treated with high-fiber or laxatives,^[6,7]^ while patients with severe FC need special cares and aggressive therapies. Several pharmacological therapeutics have been approved for FC, including diphenyl mechanics or derivatives, anthraquinone, 5-hydroxytryptamine receptor 4 (5-HT4) agonist, chloride channel type 2 activator, guanylate cyclase C receptor agonist, apical sodium bile acid inhibitors.^[8–11]^

Due to the current unsatisfactory effect of long-term use of laxatives, increasing scholars studied the efficacy and mechanism of traditional Chinese medicine to relieve constipation and achieved certain achievements.^[12]^ Certain herbs or herbal formulas have been widely used to treat FC in East Asia. Especially in China, more and more patients with FC are more willing to seek help from traditional Chinese medicine.^[13]^ Furthermore, studies proved that Qi Di Laxative Decoction was an effective treatment way for FC. However, its safety and effectiveness should be verified by further studies.

This review aims to systematically review all randomized controlled trials (RCTs) to assess the clinical efficacy of Qi Di Laxative Decoction for patients with FC.

## 2 Materials and methods

This systematic review protocol has been registered on OSF (Registration number: DOI 10.17605/OSF.IO/M2ESR). The protocol follows the Cochrane Handbook for Systematic Reviews of Interventions and the Preferred Reporting Items for Systematic Reviews and Meta-Analysis Protocol statement guidelines.^[14]^ We will describe the changes in our full review if needed.

## 3 Inclusion criteria for study selection

### 3.1 Type of studies

This review will include clinical RCTs of Qi Di Laxative Decoction for FC (FC) patients without any language or publication status restrictions. Non-RCTs, quasi-RCTs, case series, case reports, crossover studies, uncontrolled trials, and laboratory studies will not be included.

### 3.2 Type of participants

Participants who were diagnosed with FC according to related guidelines or consensus. All included participants in this review regardless of their age, race, and gender.

### 3.3 Type of interventions

Qi Di Laxative Decoction are given to the treatment group and placebo or another treatment has been compared with Qi Di Laxative Decoction. Control: no intervention, treatments other than Qi Di Laxative Decoction (e.g., usual or standard care, placebo, wait-list controls).

### 3.4 Type of outcome measures

#### 3.4.1 Main outcome(s)

The main outcomes will include overall effective rate, recovery rate, and colonic transmission function.

#### 3.4.2 Additional outcome(s)

The secondary outcomes will include symptom improvement, recurrence rate, quality of life score, and intestinal flora level.

## 4 Search methods for the identification of studies

### 4.1 Electronic searches

We will search the following electronic bibliographic databases for relevant trials:

China National Knowledge Infrastructure Database (from 1979–present);
Wanfang Database (from 1990–present);
Pubmed Database (from 2000–present);
Cochrane Central Register of Controlled Trials (from 2000–present);
Cumulative Index of Nursing and Allied Health Literature (from 1937–present);
Excerpta Medica database (from 1947–present);
Ovid MEDLINE ALL (Ovid Medical Literature Analysis and Retrieval System Online, from 1946–present);
In addition, Clinical trial registries, like the Chinese Clinical Trial Registry (ChiCTR), the Netherlands National Trial Register (NTR) and ClinicalTrials.gov, will be searched for ongoing trials with unpublished data.

There will be no language restrictions.

### 4.2 Data collection and analysis

#### 4.2.1 Study identification

We will use EndNote X9 software (Alfasoft Limited, A.W. house, United Kingdom) to manage the records of searched electronic databases. The initial selection will involve scanning of the titles and abstracts of the retrieved studies. The full text of relevant studies will then be reviewed for study inclusion, in accordance with the inclusion criteria, by 2 authors. Potentially relevant articles will be reviewed independently by 2 authors to determine if they meet the prespecified criteria. Any disagreement between authors will be resolved by consensus with a third author. The study selection procedure will follow and be recorded in the Preferred Reporting Items for Systematic Reviews and Meta-analysis flow chart. All the evidence will be assessed by The Grading of Recommendations Assessment, Development and Evaluation.

#### 4.2.2 Data extraction and management

According to the inclusion criteria, a standard data collection form will be made before data extraction. The following data will be extracted by 2 authors:

General information: research identification, publication year, the title of the study, first author;
Study methods: study design, sample size, randomization method, allocation concealment, blinding, incomplete report or selecting report, other sources of bias;
Participants: Inclusion and exclusion criteria;
Intervention: motion details, treatment duration, and frequency;
Control: Type of control methods, motion details, treatment duration, and frequency;
Outcomes: Included outcome measures.

#### 4.2.3 Risk of bias assessment.

The risk of bias in included studies will be assessed independently by 2 reviewers using the Cochrane Risk of Bias Tool, with any disagreements resolved by consensus or by discussion with a third reviewer. All judgments will be fully described, and the conclusions will be presented in the Risk of Bias figures and will be incorporated into the interpretation of review findings, by means of sensitivity analysis. The risk of bias of each domain will be graded as adequate, unclear, or inadequate. We intend to use the concealment of allocation grading in investigation of any heterogeneity and in sensitivity analysis. Other aspects of study quality including the extent of blinding (if appropriate), losses to follow up, non-compliance, whether the outcome assessment was standardized, and whether an intention to treat analysis was undertaken, will be presented in the risk of bias table describing the included studies and will provide a context for discussing the reliability of the results.

#### 4.2.4 Data analysis

We will use Stata Software (StataCorp: College Station, TX, USA) [Computer program] (Version 15.1) to process the meta-analysis. Weighted mean difference will be used for continuous variable data, and the combined statistical effects of these 2 are combined. The X^2^ test will be adopted to analyze whether there is heterogeneity in each of the included research questions. I^2^ > 50% is a criterion for significant judgment. The fixed effect model is adopted if I^2^≤50%, which is considered to have homogeneity between the studies. The random effect model is adopted if I^2^ > 50%, which is considered to have heterogeneity among the studies. The effect size is expressed as 95% confidence interval, and *P* < .05 is considered to be statistically significant.

##### 4.2.4.1 Sensitivity analyses

Heterogeneity may be due to the presence of 1 or more outlier studies with results that conflict with the rest of the studies. We will perform sensitivity analyses excluding outlier studies. In addition, we plan to perform sensitivity analysis to explore the influence of trial quality on effect estimates. The quality components of methodology include adequacy of generation of allocation sequence, concealment of allocation, and the use of intention-to-treat analysis.

##### 4.2.4.2 Meta-Regression analyses

If data permits, we will perform the meta-regression analyses.

#### 4.2.5 Publication bias

If sufficient number of trials (more than 10 trials) are found, we will generate funnel plots (effect size against standard error) to investigate publication bias.

#### 4.2.6 Ethics and dissemination

The data used in this systematic review will be collected from published studies. Based on this, the study does not require ethical approval.

## Acknowledgments

The authors would like to thank all the researchers in our working group.

## Author contributions

**Conceptualization:** Jianfang Wang, Zhongmei Sun.

**Data curation:** Jian-Fang Wang, Zhong-Mei Sun.

**Formal analysis:** Zhongmei Sun.

**Funding acquisition:** Jianfang Wang.

**Methodology:** Jian-Fang Wang, Zhong-Mei Sun, Jun-Xiang Li.

**Resources:** Jian-Fang Wang.

**Software:** Jian-Fang Wang, Zhong-Mei Sun.

**Supervision:** Jun-Xiang Li.

**Writing – original draft:** Jian-Fang Wang, Zhong-Mei Sun.

**Writing – review & editing:** Jian-Fang Wang, Zhong-Mei Sun, Jun-Xiang Li.
